# Psychotherapeutic Treatment for Anorexia Nervosa: A Systematic Review and Network Meta-Analysis

**DOI:** 10.3389/fpsyt.2018.00158

**Published:** 2018-05-01

**Authors:** Almut Zeeck, Beate Herpertz-Dahlmann, Hans-Christoph Friederich, Timo Brockmeyer, Gaby Resmark, Ulrich Hagenah, Stefan Ehrlich, Ulrich Cuntz, Stephan Zipfel, Armin Hartmann

**Affiliations:** ^1^Department of Psychosomatic Medicine and Psychotherapy, Faculty of Medicine, Medical Center–University of Freiburg, University of Freiburg, Freiburg, Germany; ^2^Department of Child and Adolescent Psychiatry, Psychosomatics and Psychotherapy, University Hospital of the RWTH Aachen, Aachen, Germany; ^3^Department of Psychosomatic Medicine and Psychotherapy, Medical Faculty, Heinrich Heine University, Düsseldorf, Germany; ^4^Department of Psychosomatic Medicine and Psychotherapy, University Hospital Tuebingen, Tuebingen, Germany; ^5^Division of Psychological and Social Medicine and Developmental Neurosciences, Department of Child and Adolescent Psychiatry, Faculty of Medicine, Technische Universitaet Dresden, Dresden, Germany; ^6^Schön Klinik Roseneck, Prien am Chiemsee, Germany

**Keywords:** anorexia nervosa, eating disorders, systematic review, psychotherapy, meta-analysis

## Abstract

**Background:** The aim of the study was a systematic review of studies evaluating psychotherapeutic treatment approaches in anorexia nervosa and to compare their efficacy. Weight gain was chosen as the primary outcome criterion. We also aimed to compare treatment effects according to service level (inpatient vs. outpatient) and age group (adolescents vs. adults).

**Methods:**The data bases PubMed, Cochrane Library, Web of Science, Cinahl, and PsychInfo were used for a systematic literature search (until Feb 2017). Search terms were adapted for data base, combining versions of the search terms anorexia, treat^*^/therap^*^ and *controlled trial*. Studies were selected using pre-defined in- and exclusion criteria. Data were extracted by two independent coders using piloted forms. Network-meta-analyses were conducted on all RCTs. For a comparison of service levels and age groups, standard mean change (SMC) statistics were used and naturalistic, non-randomized studies included.

**Results:** Eighteen RCTs (trials on adults: 622 participants; trials on adolescents: 625 participants) were included in the network meta-analysis. SMC analyses were conducted with 38 studies (1,164 participants). While family-based approaches dominate interventions for adolescents, individual psychotherapy dominates in adults. There was no superiority of a specific approach. Weight gains were more rapid in adolescents and inpatient treatment.

**Conclusions:** Several specialized psychotherapeutic interventions have been developed and can be recommended for AN. However, adult and adolescent patients should be distinguished, as groups differ in terms of treatment approaches considered suitable as well as treatment response. Future trials should replicate previous findings and be multi-center trials with large sample sizes to allow for subgroup analyses. Patient assessment should include variables that can be considered relevant moderators of treatment outcome. It is desirable to explore adaptive treatment strategies for subgroups of patients with AN. Identifying and addressing maintaining factors in AN remains a major challenge.

## Introduction

The treatment of anorexia nervosa (AN) is one of the most challenging, with psychotherapy being considered the primary intervention (1–3). Anorexia nervosa affects predominantly young females and leads to significant impairment in health and functioning (3). It shows a chronic course (4) and is associated with a high mortality rate as well as considerable burdens for individuals, families, and society (5). Although progress has been made in the understanding of psychosocial and biological mechanisms that are responsible for the development of the illness and its maintenance, there is still an urgent need to optimize treatment approaches and demonstrate their efficacy. The psychotherapeutic approaches that have been developed for the treatment of anorexia nervosa so far were traditionally oriented on a cognitive-behavioral model, a family systems model or a psychodynamic model (see for example (6, 7)). Most recent approaches try to integrate and address new empirical findings like the relevance of cognitive inflexibility in AN (8).

In a previous review for the first version of the German treatment guidelines for eating disorders including publications until August 2008, 57 studies on psychotherapeutic treatments (randomized controlled trials as well as naturalistic studies) could be identified and were included in a meta-analysis of standardized mean change (9). The studies included 84 treatment arms with 2,273 patients. Randomized-controlled studies up to this point had small sample sizes and poor methodological quality, therefore naturalistic studies were included when fulfilling specific inclusion criteria. Results did not point to the superiority of one treatment over another. Overall, weight gain was higher in an inpatient setting compared to outpatient treatment (531 vs. 262 g/week), when considering the development over a period of 26 weeks (9). In the following years, studies improved considerably in methodological quality (1, 3). Furthermore, a Cochrane review on individual outpatient treatment in adult anorexia nervosa was conducted in the meantime (10). This review included 10 studies. However, the studies entailed trials of very low methodological quality (e.g., with a sample size of *N* < 10 per treatment arm and studies that evaluated interventions focusing on only a specific aspect of AN symptomatology like cognitive flexibility (11). A Cochrane Review (12) on family therapy included studies until January 2008.

### Research question

For a revision of the German treatment guidelines for treatment of eating disorders (13), we aimed to systematically review the literature during the last 9 years, updating our initial meta-analysis (9). The research questions for this study were: (1). What is the recent evidence base for psychotherapeutic treatment in anorexia nervosa (AN)? (2). What is the comparable effectiveness of different treatments? (3). What is the amount of weight gain that can be expected by interventions at different service levels and in different age groups (adolescents vs. adults)?

Psychotherapy was defined as a treatment that uses psychological methods in direct personal contact between a patient and a therapist with the aim of overcoming mental illness.

## Methods

### Search strategy and selection of studies

**Figure 1:**
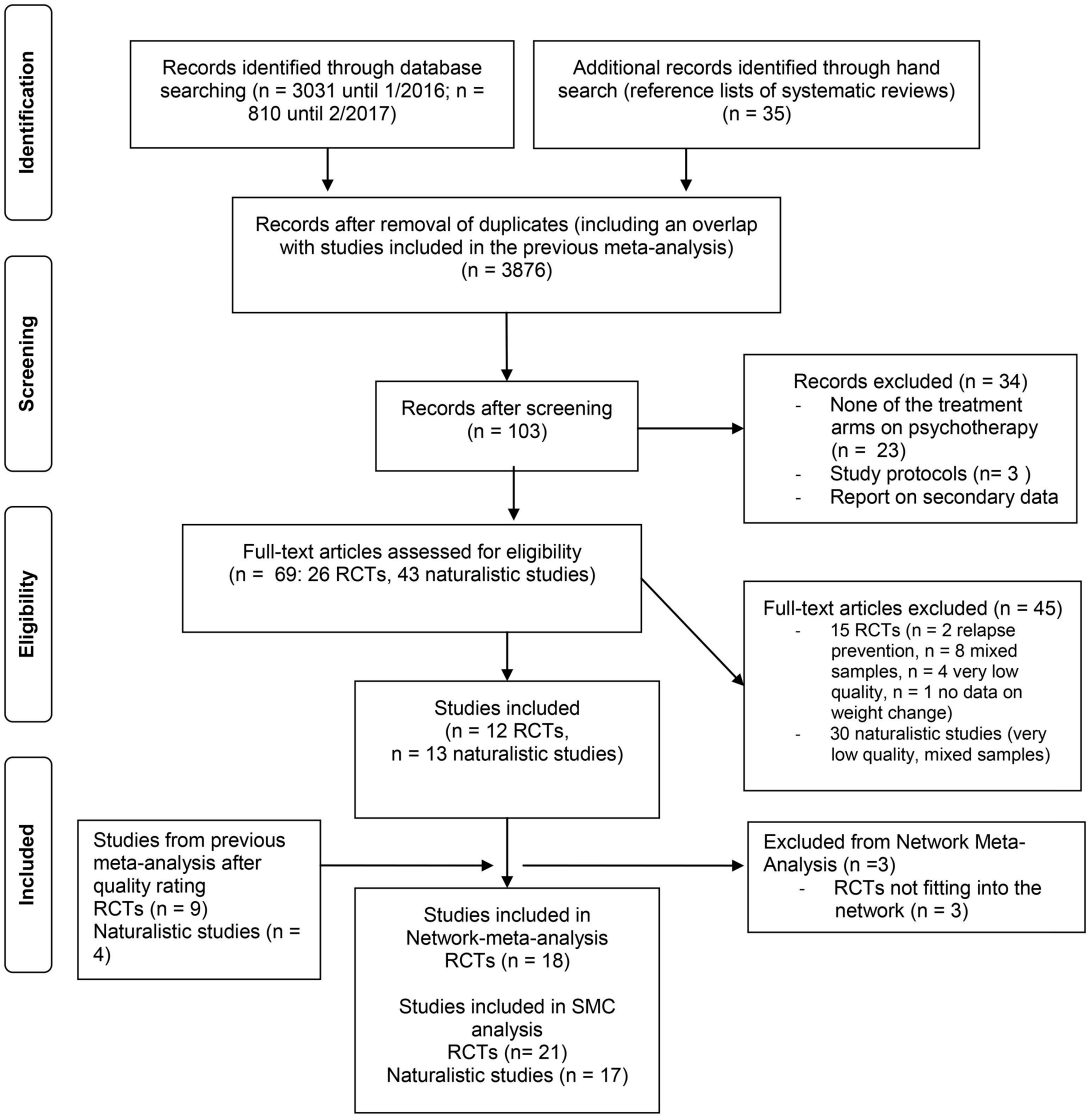
PRISMA flow chart, study selection procedure.

A systematic literature search was conducted by the University library in Heidelberg/Germany using the following data bases: PubMed, Cochrane Library, Web of Science, Cinahl, PsychInfo, ClinicalTrial.gov and ICTRP^1^, including all publications until February 2017 using a complex search strategy combining the search terms anorexia, treat^*^/therap^*^ and *controlled trial* (complete search strategy: http://www.awmf.org/leitlinien/detail/ll/051-026.html). The abstracts of publications were screened and all studies were excluded that did not focus on psychotherapeutic interventions in AN or were written in languages other than English or German (PRISMA flow chart: see Figure 1) (14). In a second step, all articles were excluded which were study protocols, that reported on secondary data analyses or that did not aim to assess psychotherapeutic interventions, resulting in a number of 26 RCTs and 44 naturalistic studies published since the previous meta-analysis. Each study was rated by two independent coders. In the case of a disagreement, the publication was checked again and a consensus was found after discussion. A final selection was conducted according to the inclusion criteria used in the first meta-analysis, adding one additional criterion: the quality of a study. Inclusion criteria were the following:

- At least one treatment arm included a psychotherapeutic intervention
- Data for body weight/body mass index are reported for at least two time points of measurement
- The sample size of the whole study is greater or equal to N_arms_ × 10 (e.g., 11 + 9 = 20; ≥10 × 2) for RCTs and >30 per treatment arm in naturalistic studies
- The sample consists solely of patients with anorexia nervosa, or results for the subsample of patients with anorexia nervosa are reported separately
- The second time point of measurement has to be within a time frame of 3 years after the beginning of treatment (so that results can be attributed to the intervention)
- Studies are of high, moderate, or low quality (studies of very low quality were excluded).

All studies (including the studies from the previous meta-analysis) were additionally rated in terms of quality. The criteria used were the following and rated with “yes” or “no” for each study: (1). Sample size > 30/arm, (2). Recruitment bias (e.g., inclusion and exclusion criteria: sample representative for the population of interest?), (3). Drop-out-rate < 20%, (4). Intention-to-treat (ITT)-analysis, (5). All relevant outcomes reported (weight and eating pathology), (6). Use of validated outcome measures, (7). Allocation concealment, (8). Blinding, (9). Consort statement (Consort = Consolidated Standards of Reporting Trials), (10). Registered in trial register, (11). Population of interest, (12). Intervention of interest, (13). Endpoints clinically relevant, (14). Intervention can be implemented and used in the German health care system, (15). Acceptability (criteria 7, 9, and 10 were only used for RCTs). Based on these criteria, each study was rated as being of high, moderate, low or very low overall quality. If raters differed in their quality rating, they found a consensus after discussion. Studies were categorized as “very low quality” when they did not meet the criteria 4, 6, 8, and 9. For more details on quality ratings see Supplement 1.

In order to address the third research question, we included naturalistic studies (non-randomized trials, observational studies) in the analyses, like in the previous meta-analysis. The rationale behind this decision was to increase external validity for a comparison of weight gain in different service levels and age groups.

Data was extracted from the studies by the two independent coders using piloted forms, checked for congruence and finally entered into an MS-Access data base.

For included studies see Table 1, for excluded studies see Supplement 2.

**Table 1:** Included studies.

**References**	**Follow-up week**	**Trt arm**	**N**	**N drop out**	**Setting**	**TrtType**	**Study quality**
**(A) RCTs ADOLESCENT SAMPLES**							
Agras et al. (38)	88	1	78	20	Outpatient	FT_AN	Moderate
		2	80	20	Outpatient	FST	
Eisler et al. (39)	52	1	21	2	Outpatient	FT_AN sep.	Moderate
		2	19	2	Outpatient	FT_AN conj.	
Eisler et al. (40)	52	2	86	9	Outpatient	MFT	High
		1	83	9	Outpatient	FT_AN	
Gowers et al. (41)	52	1	55	17	Outpatient	Complex-op ^°^	Moderate
		2	55	14	Outpatient	FT_AN&X ^*^	
		3	57	29	Inpatient	Complex-ip	
Herpertz-Dahlmann et al. (42)	68	1	85	10	Inpatient	Complex-ip	High
		2	87	25	Day hospital	Compex-dh	
Le Grange et al. (43)	52	1	55	9	Outpatient	FT_AN conj.	Moderate
		2	52	8	Outpatient	FT_AN sep.	
Lock et al. (44)	52	1	44	7	Outpatient	FT_AN	High
		2	42	10	Outpatient	FT_AN&X ^#^	
Lock et al. (45)	52	1	60	4	Outpatient	PD&X	Moderate
		2	61	13	Outpatient	FT_AN	
Madden et al. (46)	52	1	41	5	In/outpatient	Complex-ip short^*^	Moderate
		2	41	8	Inpatient	Complex-ip	
Robin et al. (47)	63.6	1	19	1	Outpatient	FT_AN	Low
		2	18	1	Outpatient	PD&X	
**(B) RCTs ADULT SAMPLES**							
Crisp et al. (35)^**^	104	2	20	2	Outpatient	Complex-op^°°^	Moderate
		4	20	0	Outpatient	TAU	
Dalle Grave et al. (37)^*^	76	1	42	5	Inpatient	Complex-ip	High
		2	38	3	Inpatient	Complex-ip&X ^##^	
Dare et al. (48)	52	1	19	7	Outpatient	FPT	Low
		2	21	5	Outpatient	FT_AN	
		3	22	9	Outpatient	CAT	
		4	17	4	Outpatient	TAU	
Hall et al. (36)		1	15	1	Outpatient	Complex-op ^°°°^	Low
		2	15	4	Outpatient	Diet&X	
Lock et al. (11)	24	1	23	3	Outpatient	CBT&X^##^	Moderate
		1	23	8	Outpatient	CBT	
McIntosh et al. (49)	20	1	19	7	Outpatient	CBT	Moderate
		2	21	9	Outpatient	IPT	
		3	16	5	Outpatient	SSCM	
Schmidt et al. (8)	52	1	72	18	Outpatient	MANTRA	High
		2	70	29	Outpatient	SSCM	
Schmidt et al. (50)	52	1	34	10	Outpatient	MANTRA	High
		1	37	16	Outpatient	SSCM	
Touyz et al. (51)	56	1	31	1	Outpatient	CBT	High
		2	32	2	Outpatient	SSCM	
Treasure et al. (52)	52	1	16	6	Outpatient	CBT	Moderate
		2	14	4	Outpatient	CAT	
Zipfel et al. (53)	52	1	80	8	Outpatient	FPT	High
		2	80	17	Outpatient	CBTE	
		3	82	29	Outpatient	TAU	
**(C) NATURALISTIC STUDIES ADOLESCENT SAMPLES**							
Dalle Grave et al. (54)	100	1	46	17	Outpatient	CBTE	Low
Dalle Grave et al. (55)	100	1	27	1	Inpatient	Complex-ip	Low
Herpertz-Dahlmann et al. (56)	108	1	39	?	Inpatient	Complex-ip	Low
Schlegl et al. (57)	11.7	1	262	47	Inpatient	Complex-ip	Moderate
**(D) NATURALISTIC STUDIES ADULT SAMPLES**							
Abbate-Daga et al. (58)	72	1	56	6	Day Hospital	Complex-dh	Moderate
Bowers et al. (59)	33	1	32	?	Inpatient	Complex-ip	Low
Channon et al. (60)	61	1	45	?	Inpatient	Complex-ip	Low
Fairburn et al. (61)	100	1	50	19	Outpatient-GB	CBTE	Moderate
		2	49	17	Outpatient-I	CBTE	
Fichter and Quadflieg (62)	104	1	103	?	Inpatient	Complex-ip	Low
Fittig et al. (63)	20	1	100	26	Day clinic	Complex-dh	Low
Goddard et al. (64)	26.4	1	150	?	Inpatient	Complex-ip	Moderate
Kohle et al. (65)	260	1			Inpatient	Complex-ip	Low
Long et al. (66)	208	1	34	5	Inpatient	Complex-ip	Moderate
Ricca et al. (67)	40	1	53	10	Outpatient	CBT	Moderate
Treat et al. (68)	4.8	1	73	2	Inpatient	Complex-ip	Low
Wade et al. (69)	72	1	28	5	Outpatient	MANTRA	Low
Willinge et al. (70)	4.7	1	33	8	Day hospital	Complex-dh	Low

*Classification of treatments in some cases had to be adapted to specific circumstances of the method and the sample of included studies: For example, there are studies comparing variants of a specific treatment, e.g., various forms of family-based treatment as a short or long term intervention or seeing the whole family vs. parents and patient separately (39, 44). In these cases, we identified the most typical treatment arm for a treatment class (e.g., psychodynamic therapy PD) and labeled the other(s) as its variant by adding “&X” (e.g., PD&X). Inpatient and day hospital programs as well as outpatient interventions entailing a broad range of treatment elements were labeled “complex” treatments*.

CBTE, cognitive-behavior therapy enhanced; CBT, cognitive behavior therapy; MFT, multi family therapy; FT_AN, family based treatment for anorexia nervosa; FST, Family systems therapy; MANTRA, Maudsley Model of Anorexia nervosa Treatment for Adults; IPT, Interpersonal Psychotherapy; SSCM, Specialist Supportive Clinical Management; CRT, Cognitive Remediation Therapy; FPT, Focal Psychodynamic Psychotherapy; CAT, Cognitive-Analytic Therapy; PD, Psychodynamic Therapy; complex, several treatment components; -ip, inpatient; -dc, day clinic; -op, outpatient; sep., separate (familiy therapy); conj., conjoint (family therapy); diet, dietary advice; GB, Great Britain; I = Italy;

# FT_AN in a short version was labeled as a variant: FT_AN&X;

## CBT-E in an inpatient setting in a focussed (CBT-Ef) and a more “broad” form (CBT-Eb) were compared (addessing additional problems like mood intolerance and perfectionism);

* This arm was labeled “treatment as usual in the general community,” but was family-based treatment combined with dietary advice, individual supportive sessions and medical management;

** Two arms of the study could not be included, as no follow-up data were reported;

° CBT + parental feedback and counselling + dietary advice;

°° Individual + family sessions (psychodynamic orientation);

°°° Individual sessions (psychodynamic orientation) + family session + dietary advice;

### CBT&X consisted of 8 initial sessions of CRT (Cognitive Remediation Therapy) plus CBT; “ = inpatient treatment only until medical stabilization.

*TAU (treatment as usual) in the study of Dare et al. (48) was low-contact supportive out-patient management by psychiatric trainees; TAU in the study of Crisp et al. (35) was labeled “no treatment,” but consisted in management by the local family doctor or consultant who got detailed advice (patients got a range of different interventions); TAU in the study of Zipfel et al. (53), labeled “optimized treatment as usual,” was the referral of patients to experienced outpatient psychotherapists and their family doctors who got advice for medical management (overall, patients received a comparable number of psychotherapy sessions as in FPT and CBT-E) N drop out, drop out from therapy; ?, no data or only study drop outs reported*.

### Data analysis

We conducted a network-meta-analysis of all randomized trials (research questions one and two). As there are multiple treatments available for AN with only few replications of treatment comparisons, we choose the methodology of network meta-analysis to summarize the available evidence. Studies on adult and adolescent samples were analyzed separately, as both groups differ in terms of duration of illness and the state of psychological as well as physical development. For research question three, we used standardized mean change statistics according to our previous approach (9). As the main outcome criterion, we chose weight gain/change in BMI (kg/m^2^). A change in weight is the agreed upon most relevant criterion for outcome in anorexia nervosa and reported in most of the studies (15). Concerning further possible criteria (e.g., drive for thinness, body image disturbance, quality of life, cognitive flexibility), studies differed considerably in the instruments used and outcomes reported, making it impossible to choose them as secondary outcomes in a meta-analysis. Additionally, a meta-analytic summary of dropout rates could not be conducted for the following reasons: In some studies there was a major impact of the study design on drop-out rates (e.g., inpatient treatment episodes if patients lost weight), health care systems differ considerably in the availability of alternative treatments after dropping out (with a possible impact on outcome at the time point of follow up) and some studies reported study drop outs only.

### Network meta-analysis

Network meta-analysis combines direct and indirect treatment comparisons. While standard meta-analysis summarizes direct treatment comparisons only, network meta-analysis assumes transitivity. For example, study 1 shows treatment A > B_1_, and study 2 shows treatment B_2_ > C, then B (given B_1_ = B_2_) links both studies and it is assumed that A > C. For statistical assumptions and computational background see recent overviews of the method (e.g., (16, 17)). Treatments of the same type were realized at different centers by different research teams, therefore we choose random-effect models to calculate effect sizes. Statistical software to compute network meta-analyses has greatly improved (17) and is easily accessible (e.g., the *netmeta* package for R (18)).

First, the treatment arms of RCTs published over 3 decades had to be classified according to the interventions used. As approaches changed over time, this classification can only be an approximation. We decided to orient on the categorization used by Zipfel et al. (1) and Espie and Eisler (19) (see Table 1). However, the classification of family based treatments was a major challenge. We tried to summarize approaches that orient to the Maudsley model under “FT-AN,” differentiating it from family systems therapy and multi-family therapy.

The representation of standardized mean differences (SMDs) in forest plots needs a comparison treatment to contrast with all other treatments. Due to ethical reasons, placebo control groups are not available and the Treatment as Usual (TAU) conditions appear to be very heterogonous. Therefore we chose the most evaluated and manualized treatment for the central comparison position of the respective network of adult and adolescent samples: Specialist Supportive Clinical Management (SSCM) in the network of adult samples and family based treatment (FT_AN) in the adolescent network. The structures of the two networks are evaluated by distance matrices, net graphs and measures of network inconsistency (*I*^2^, tau^2^, and *Q*; see Schwarzer et al., chapter 8 (18).

### Standardized mean change (SMC) statistics

SMC standardizes the difference between two time points to the standard deviation of the first (20). The first time point was defined as the beginning of treatment for all studies. The second time point preferably was a 1-year follow-up. This is the time point that was reported in most of the studies. There seems to be a consensus that the restoration of weight in anorexia nervosa needs time and that relapses/longer term stability of the outcome are best captured after 1 year (21). If no 1-year follow up was available, we selected the next closest time point that was reported (see Table 1).

The two approaches provide different information. The network meta-analysis compares treatment effects at follow-up, assuming successful randomization at baseline. SMC statistics standardize the intake follow-up change within a single treatment, assuming a comparable amount of “spontaneous remission” over time. The effect sizes will be labeled SMC (standardized mean Change) for the intake follow-up calculations and SMD (standardized mean Difference) for the network meta-analysis (18).

## Results

### Randomized controlled studies, studies included in the network meta-analysis

Overall, 26 RCTs on psychotherapeutic treatment in AN were published since August 2008. Out of these, 6 studies were on mixed samples of eating disorder (22–27). Seven further studies had to be excluded because they were of very low quality, the design could not be compared with other studies (28–31), they addressed relapse prevention (32, 33) or they did not report data on weight change (34).

A rating of study quality of the RCTs from the previous meta-analysis revealed that 9 out of 23 studies were of sufficient quality and fulfilled all inclusion criteria. Together with the recent search, this resulted in a number of 21 RCTs (see Figure 1), of which three further studies (35–37) had to be excluded from the network meta-analysis in adults, as their treatment arms could not be classified to match any treatment category of the larger network.

Of the 18 RCTs which could finally be included in the network meta-analysis, 10 studies were on adolescents and 8 studies were on adults. It is important to note that studies with samples entailing adolescents as well as adults (11) were added to the adult subsample, if the majority of patients had an age above 18.

Weighted mean age at intake was *M* = 26.2 years for studies including adult patients (range of means: 19.6–33.6), and *M* = 15.2 (range of means: 14.3–15.7) for studies on adolescents. Five of the eighteen RCTs included only females (range of female participants in the other studies: 87.7–97.9%).

### Naturalistic studies, studies included in SMC analysis

Out of 43 naturalistic studies from the recent literature search, 13 studies fulfilled the above-mentioned criteria, as well as 4 out of 45 naturalistic studies from the previous search.

Overall, SMC analyses were conducted with 38 studies (21 RCTs, 17 naturalistic studies) comprising 18 treatment arms and 1,164 patients (4 studies on adolescents with 4 arms and 350 patients). See Supplement 2 for excluded studies.

### Network meta-analysis: recent evidence base and comparison of treatments

Several psychotherapeutic treatment approaches exist for AN. In all studies, active treatments were compared with each other. No study compared active treatments with an untreated control group.

In studies on adults, a range of different interventions—predominantly on an individual basis—were evaluated. However, only few comparisons of specific treatment approaches were replicated (see Table 1). Effect sizes of direct comparisons of treatments for adult samples are shown in Table 2.

**Table 2:** Direct comparisons between treatments.

**Comparison**	**SMD**	**seSMD**	**Study**	**Treatment 1**	**Treatment 2**	**N1**	**M1**	**SD1**	**N2**	**M2**	**SD2**	**Metric**
**(A) ADOLESCENT PATIENTS**												
1	−1.327	0.177	Agras2014	FST	FT_AN	78	92.3	9.3	78	94.6	9.3	%iBW
2	0.221	0.153	Eisler2016	MFT	FT_AN	86	90.7	6.3	86	89.3	6.3	%mBMI
3	−0.713	0.328	Eisler2000	FT_ANsep	FT_AN	19	45.7	6.6	21	50.5	6.6	kg
4	0.167	0.196	Gowers2007	Complex-op	Complex-ip	52	17.9	2.37	52	17.5	2.37	BMI
5	−0.167	0.198	Gowers2007	Complex-op	FT_AN&X	52	17.9	2.37	50	18.3	2.37	BMI
6	0.335	0.200	Gowers2007	FT_AN&X	Complex-ip	50	18.3	2.37	52	17.5	2.37	BMI
7	−0.167	0.158	HerpertzDa2014	Complex-ip	Complex-dh	75	17.8	1.5	86	18.1	2	BMI
8	−0.206	0.194	LeGrange2016	FT_AN	FT_AN&X	55	92.8	9.8	52	95	11.4	%mBMI
9	0.000	0.216	Lock2005	FT_AN&X	FT_AN	42	19.5	2.1	44	19.5	2.2	BMI
10	−0.092	0.208	Lock2010	PD&X	FT_AN	49	93.1	13.7	44	94.2	9.5	%eBW
11	0.294	0.208	Madden2015	Complex-ipS	Complex-ip	56	95.5	6.7	40	93.6	6	%aBW
12	0.841	0.449	Robin1994	FT_AN	PD&X	11	20.1	1.1	11	19	1.4	BMI
**(B) ADULT PATIENTS**												
1–6^*^	0.000^*^	0.305	Dare2001	FPT, FT_AN,CAT^*^		21-23	16.5	2.4	19-22	16.5	2.4	BMI
7	−0.737	0.306	Lock2013	CBT&X	CBT	23	17.6	1.2	23	18.5	1.2	BMI
8	0.000	0.317	McIntosh2005	CBT	IPT	19	18.1	2.47	21	18.1	2.47	BMI
9	−0.277	0.341	McIntosh2005	CBT	SSCM	19	18.1	2.47	16	18.8	2.47	BMI
10	−0.277	0.334	McIntosh2005	IPT	SSCM	21	18.1	2.47	16	18.8	2.47	BMI
11	0.493	0.270	Schmidt2012	MANTRA	SSCM	30	17.8	0.4	27	17.6	0.4	BMI
12	−0.745	0.197	Schmidt2015	MANTRA	SSCM	60	18.4	0.4	51	18.7	0.4	BMI
13	−0.127	0.252	Touyz2013	CBT	SSCM	31	16.6	1.4	32	16.8	1.7	BMI
14	−0.408	0.370	Treasure1995	CBT	CAT	16	17.4	3	14	18.5	2.1	BMI
15	0.100	0.157	Zipfel2014	CBTE	TAU	80	17.7	1	83	17.6	1	BMI
16	−0.100	0.158	Zipfel2014	FPT	CBTE	80	17.6	1	80	17.7	1	BMI
17	0.000	0.157	Zipfel2014	FPT	TAU	80	17.6	1	83	17.6	1	BMI

For abbreviations of types of treatment see Table 1;

* Dare2011 reported the grand mean only as the groups did not differ significantly. Therefore we report only one SMC for all six comparisons of the study; SMD, Standarized Mean Difference; seSMD, standard error; study, ID of main publication; N1, N2, respective sample sizes; descriptive statistics of weight variable: M1, mean tx1; M2, mean tx2; SD1, standard deviation tx1; SD2, standard deviation tx2; Metric: %aBW, Percent average Body Weight; %eBW, Percent expected body weight; %iBW, % ideal body weight; %mBMI, % mean BMI; Complex-ipS, Complex-ip “short.”

**Figure 2:**
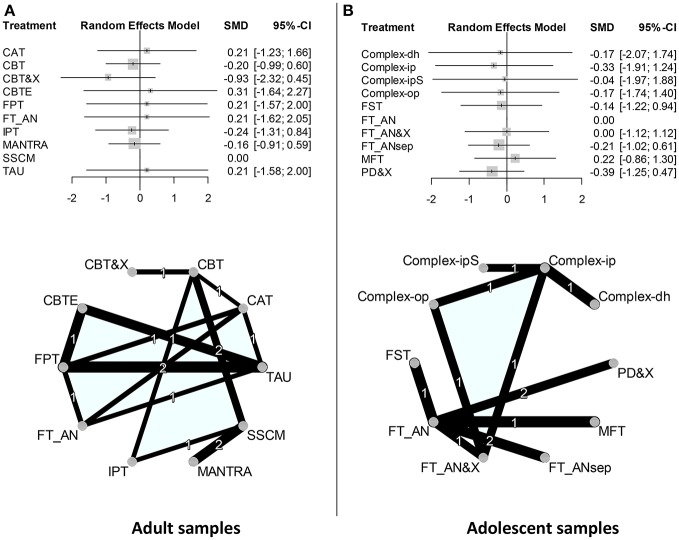
Forest plots and graphs of network meta-analyses. **(A)** Forest plot adult samples: SSCM was chosen as the reference treatment. Random effects model. If the 95%-CI includes Zero, then the SMD is not significantly different from Zero. No significant effects. Forst plot adolescent samples: FT_AN was chosen as the reference treatment. Random effects model. No significant effects. Complex-ipS = Complex-ip, “short” inpatient treatment. Net adult samples: All treatment categories are located on a circle in alphabetical order (counterclockwise, starting with CAT). All direct comparisons are represented by a connecting line. Only three direct comparisons were investigated more than once. The thickness of a connecting line is proportional to 1/SE of the respective SMD. **(B)** Net adolescent samples: FT_AN was chosen as the reference treatment. Only two direct comparisons were investigated more than once.

These direct comparisons link to a network of direct and indirect comparisons that can be described by a network-graph and distance matrix (see Figure 2 and Table 3A). The network of studies (adult) comprises five studies with two treatment arms and three studies with three or four treatment arms (*k* = 8 studies, *n* = 10 treatments, *m* = 17 pairwise comparisons; *d* = 7 designs). The test of inconsistency between designs was significant (*Q* = 13.9, *df* = 3, *p* = 0.0031). The maximum distance between nodes (indirect comparisons) was maxD _CBTE−MANTRA_ = 5. Only three connections were investigated more than once.

**Table 3:** Distance matrixes.

	**Treatment**	**(1)**	**(2)**	**(3)**	**(4)**	**(5)**	**(6)**	**(7)**	**(8)**	**(9)**	**(10)**
**(A) DISTANCE MATRIX ADULT SAMPLES**											
(1)	CAT	.	1	2	2	1	1	2	3	2	1
(2)	CBT	1	.	1	3	2	2	1	2	1	2
(3)	CBT&X	2	1	.	4	3	3	2	3	2	3
(4)	CBTE	2	3	4	.	1	2	4	5	4	1
(5)	FPT	1	2	3	1	.	1	3	4	3	1
(6)	FT_AN	1	2	3	2	1	.	3	4	3	1
(7)	IPT	2	1	2	4	3	3	.	2	1	3
(8)	MANTRA	3	2	3	5	4	4	2	.	1	4
(9)	SSCM	2	1	2	4	3	3	1	1	.	3
(10)	TAU	1	2	3	1	1	1	3	4	3	.
**(B) DISTANCE MATRIX ADOLESCENT SAMPLES**.											
(1)	Complex-dh	.	1	2	2	4	3	2	4	4	4
(2)	Complex-ip	1	.	1	1	3	2	1	3	3	3
(3)	Complex-ipS	2	1	.	2	4	3	2	4	4	4
(4)	Complex-op	2	1	2	.	3	2	1	3	3	3
(5)	FST	4	3	4	3	.	1	2	2	2	2
(6)	FT_AN	3	2	3	2	1	.	1	1	1	1
(7)	FT_AN&X	2	1	2	1	2	1	.	2	2	2
(8)	FT_ANsep	4	3	4	3	2	1	2	.	2	2
(9)	MFT	4	3	4	3	2	1	2	2	.	2
(10)	PD&X	4	3	4	3	2	1	2	2	2	.

*(A) k = 8 studies; n = 10 treatments; m = 17 pairwise comparisons; d = 7 designs; I^2^ = 0.8%; tau^2^ = 0.241, and Q = 13.9, df = 3, p = 0.0031. (B) k = 10 studies; n = 10 treatments; m = 12 pairwise comparisons; d = 8 designs; I^2^ = 0.8%; tau^2^ = 0.278, and Q = 8.12, df = 2, p = 0.0017*.

Results do not point to the superiority of one treatment option over another (for indirect effect sizes/forest plots see Figure 2). There was significant heterogeneity of effect sizes (*Q* = 13.867; *df* = 3; *p* = 0.003).

The network of studies (adolescent) comprises five studies with two treatment arms and three studies with three or four treatment arms (*k* = 8 studies, *n* = 10 treatments, *m* = 17 pairwise comparisons; *d* = 7 designs). The test of inconsistency between designs was significant (*Q* = 13.9, *df* = 3, *p* = 0.0031). The maximum distance between nodes (indirect comparisons) was maxD = 4. Only two connections were investigated more than once. This network comprises two weakly connected subnets (family treatment studies vs. studies on complex settings).

Trials on adolescents were dominated by different variants of family-oriented treatments in an outpatient setting (all direct comparisons of treatments are shown in Table 2, for network graph see Figure 2, for distance matrix Table 3B), with few exceptions including one large trial comparing inpatient treatment vs. a combination of initial short-term hospitalization followed by day hospital treatment (42). However, also hospital treatment in adolescents includes family-oriented interventions as an important component. Only two of the included studies on outpatients compared family-based interventions with individual psychotherapy (47, 45). In an additional analysis on these two studies, family-based interventions were slightly more effective, but without statistical significance. There are only two replications of direct comparisons.

The forest plot of effect sizes (Figure 2) shows insignificant differences. Heterogeneity of effect sizes was not significant (*Q* = 2.797; *df* = 2; *p* = 0.247).

### SMC analysis

#### Weight gain in different age groups

The SMC statistics were integrated by study type (RCT vs. naturalistic study) and age of the sample (adult vs. adolescent). The estimated mean effect sizes were higher in adolescent samples (SMC _RCTadults_ = 1.02 [CI95: 0.91;1.13], [*Q* = 81.2; *df* = 25; *p* < 0.0001] vs. SMC _RCTadolesc_ = 1.97 [CI95: 1.85;2.10], [*Q* = 69.58; *df* = 18; *p* < 0.0001] and SMC_natur_adults_ = 1.42 [CI95: 1.30;1.55], [*Q* = 92.83; *df* = 13; *p* < 0.0001] vs. SMC_natur_adolesc_ = 1.84 [CI95: 1.64;2.05], [*Q* = 19.02; *df* = 3; *p* < 0.0003]). The confidence intervals implied that the SMC of adult and adolescent treatments are differing significantly. However, the Q-Statistic was significantly different from zero in all four categories indicating a large variability of SMC within each group. Therefore, the result must be interpreted with caution.

#### Weight gain in different service levels

Finally, we aimed to calculate weight gains that can be expected in different treatment settings (see Table 4). We differentiated between estimates of weight gain in inpatient and outpatient samples for adults and adolescents for follow-ups ≤ 27 weeks and follow-ups of 27 weeks or more.

**Table 4:** Estimates of weight gain.

**Age group**	**Setting**	**Follow-up** **category**	**N** **arms**	**N** **patients**	**BMI** **at intake**	**BMI** **at first-follow-up**	**M** **weeks**	**BMI** **gain/week**	**gr/week**
Adolescents	Inpatient	Under 27weeks	3	318	15.0	17.4	9.8	0.25	615
		27 weeks plus	4	208	15.3	18.3	71.6	0.04	110
	Outpatient	Under 27 weeks	26	193	16.7	18.7	26.0	0.08	192
		27 weeks plus	11	545	15.9	18.5	52.0	0.05	126
Adults	Inpatient	Under 27 weeks	7	511	14.2	17.5	17.0	0.19	537
		27 weeks plus	na						
	Outpatient	Under 27 weeks	9	315	16.8	17.7	24.6	0.04	105
		27 weeks plus	19	664	16.1	17.4	43.3	0.03	87

*For studies reporting weight as kilogram and not providing data on height, BMIs were estimated by assuming a height of 168cm in adults and 158 cm in adolescents (otherwise, original data were used); na, not applicable, no data; m, mean; gr, grams*.

Overall, the mean BMI when starting treatment was clearly different: While inpatients started with an average BMI between 14 and 15 kg/m^2^, outpatients had an average BMI of 16 kg/m^2^ and above. For follow-ups ≤ 27 weeks, weight gains in inpatient settings were higher, with a mean weight gain of 537 g/week in adults and 615 g/week in adolescents; in an outpatient setting, weight gains were 105 g/week in adults and 192 g/week in adolescents.

## Discussion

In summary, the evidence base for psychotherapeutic treatments in AN has considerably improved with more than 20 randomized-controlled trials published since our last meta-analysis (which included studies up until August 2008). This is encouraging, as treatment studies in AN are difficult to conduct, due to the ambivalence of patients to engage in treatment. Furthermore, there is no evidence so far that pharmacotherapy is an alternative treatment option (71).

Today, a range of manualized psychotherapeutic treatments exist for AN like Focal Psychodynamic Therapy (FPT), the Maudsley Model of Anorexia Nervosa Treatment for Adults (MANTRA), Enhanced Cognitive Behavior Therapy (CBT-E), Family-based Treatment (FT_AN) or Specialist Supportive Clinical Management (SSCM). For a description of the content of these treatments we refer to Zipfel et al. (1). The treatments were evaluated in trials of high quality. These treatments for AN have the best evidence base. However, a major limitation in AN research is the lack of untreated comparison groups—a situation that also applies to other mental disorders. For ethical reasons (high mortality rate; physical risks of the disorder, danger of a chronic course leading to the recommendation to treat AN as early as possible), there are still no studies with untreated or waiting list control groups. Therefore, we do not know much about the “real” efficacy of treatments.

While the overall efficacy of new treatments can be assessed in comparison to an untreated control group, its superiority should be demonstrated against “treatment as usual” (TAU). TAU conditions are always “active treatments.” However, they differ according to the health care system in which the study was conducted and are not an ideal reference point. For example, TAU-O in the ANTOP-study (53) comprised of the optimized outpatient treatment available in the German health care system, with the consequence that AN patients randomized in this condition received as many outpatient sessions as patients in the manualized treatment arms. The treatment was conducted by experienced psychotherapists. Additionally, patients in the TAU-O condition on average had a higher number of inpatient admissions. Similar challenges were also described for meta-analyses in other mental disorders (72).

The network meta-analysis on adult studies shows that a comparison of psychotherapeutic approaches is further confounded by the fact that only few comparisons of treatments were independently replicated (the two comparisons of MANTRA vs. SSCM, for example, were conducted by the same research group).

In summary, our previous finding that there was no superiority of one psychotherapeutic treatment modality for adult AN over another was replicated (9, 10). One possible explanation could be that all specialized treatments address two important problem areas: They focus on weight and eating behavior as well as psychological problems (e.g., pathology of the self, affect regulation, dysfunctional cognitions, interpersonal difficulties).Overall, despite some large randomized-controlled trials of high quality published in the last years, the efficacy of specialized treatments for AN can only be assumed based on changes of relevant outcomes over time and the presumption that there will be no or only little change in untreated individuals.

Most recently, a revision of the NICE guidelines (UK) (73) was published (May 2017). A systematic review and extensive meta-analyses were conducted to answer several detailed questions. However, with few exceptions, only low quality evidence statements could be derived (GRADE-criteria), mostly based on one or two studies with a high risk of bias and imprecision. This is in line with our finding that the overall evidence base is sparse.

The evidence base in adolescents is clearly distinctive from the one in adults. Research is dominated by studies on family-based treatments. While this seems reasonable from a clinical perspective, methodologically it limits the number of comparisons to other approaches and therefore the validity of evidence statements. For example, there are still not enough high-powered studies to show superiority of family-based treatment over individual interventions. In the study by Lock et al. (45), for example, FBT was superior to adolescent focused individual therapy in terms of remission rates at a 6 and 12-month follow-up. However, in the follow-up (74), more patients in the individually treated group had gained additional weight and were recovered from the eating disorder than in the FBT-group. Thus, although FBT works more quickly, the individual intervention does not seem to be less effective in the long run. Overall, only 30% of the patients in the FBT group remained weight-restored at the 4-year follow-up. Research focused primarily on variants of family-based treatments such as short-term or long-term interventions, seeing patient and parents together or separately, or single vs. multi-family approaches. Moreover, most FBT-based trials were performed by the same group. Thus, the same problem emerges like in adult AN research: There is a need for an independent replication of the findings.

The ethical problems described above will remain a major challenge for further AN research. However, one road of research might be easier to follow: The identification of subgroups of patients that might benefit from one vs. another treatment approach. Findings of the study of Schmidt et al. (75) give a first hint in this direction, showing that in more severe patients MANTRA was more effective than SSCM. A similar statement is true for adolescent AN: Patients with severe obsessive-compulsive symptoms had a greater benefit from systemic family therapy than from FBT (38).

Comparing outcomes and effect sizes (SMC) in adults and adolescents, psychotherapeutic interventions in adolescents seem to be more effective—at least in terms of weight gain. Although it might be considered somewhat arbitrary to distinguish two groups since with most patients AN starts in childhood/adolescence and continues until adulthood, it seems to make sense in terms of clinical interventions and research.

In terms of weekly weight gains that can be achieved in hospital or in outpatient settings, we replicated our previous finding of a lower weekly weight gain in an outpatient setting. As we included outpatient studies of higher quality this time and differentiated between adolescents and adults, we assume that the expectation of weekly weight gains around 100 g per week in adult outpatients is more reliable compared to the data from our first publication (262 g/week). As the sample entails patients with good and patients with poor outcome, weight gains in successful treatments may be between 100 and 500 g/week. The finding for the mean weight gain in inpatient settings for adults remained nearly the same (previous publication: 531 g/week, recent finding: 537 g/week). Overall, adolescents show higher weekly weight gains, which are reflected in higher effect sizes. The initial weight and thus the symptom severity varied between different service levels. With a BMI below 16 or 15 kg/m^2^, inpatient treatment seems to be the treatment of choice. We assume that the more rapid weight gains in inpatient and day hospital treatment are due to the close monitoring of meals and eating habits in these settings as well as to a better containment of the anxiety caused by weight gain (holding function of a whole team).

### Limitations

**Figure 3:**
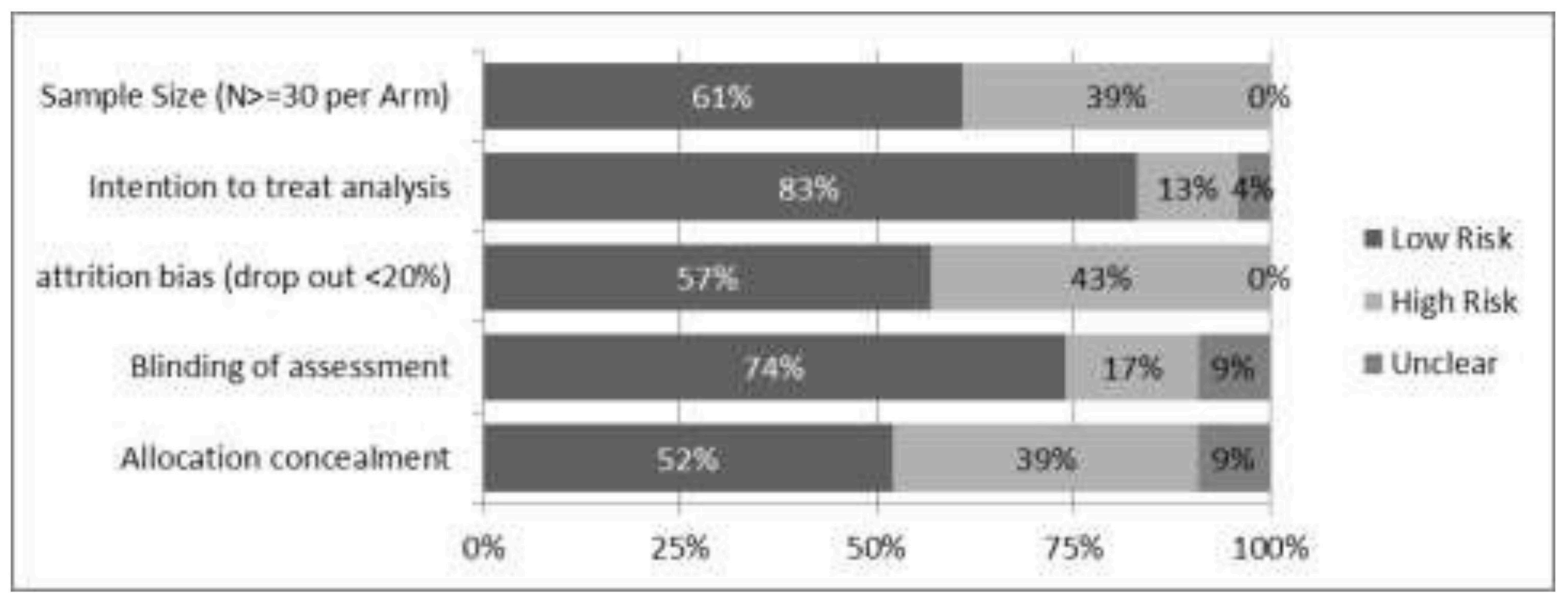
Ratings of items related to risk of bias. Risk of bias across all studies included in the network meta-analysis (coders assessment), presented as percentages of ratings (low risk: rated “yes;” high risk: rated “no”). Further possible risks of bias: Selective outcome reporting: Registration in a trial register or published study protocols were available for more recent studies only. Therefore, selective outcome reporting could not be assessed. Researcher allegiance (RA): It was taken care of that coders were independent and not involved in the studies they had to rate. The study group consisted of experts representing a broad range of therapeutic orientations (CBT, psychodynamic, family) and backgrounds (psychology, psychosomatic medicine, child, and adolescent psychiatry).

Potential modifiers of effect sizes in the network meta-analyses are differences in the samples at the beginning of treatment. For example, mean body weight of inpatient (vs. outpatient) samples at the beginning of treatment was considerably lower. There was a large variability in the kind of psychotherapeutic approaches and settings (outpatient, day hospital, inpatient, multi-family) of the included studies. The transitivity assumption of the network is rather weakly justified, as the treatment arms building the nodes between studies were not always realized as replications of manualized treatments. Especially for adolescent studies, there are only few connections between a subnet of family based treatments and a subnet of complex inpatient and day hospital treatments. In both networks only few comparisons have been studied more than once. As the Q-statistics for network inconsistency between designs rely on multiple comparisons, the statistical tests are based on a very small number of comparisons in both networks, and need to be interpreted with caution. Further, there are indirect comparisons between treatments which are separated by 4 (adolescent) or 5 nodes (adult samples). The transitivity assumption is very optimistic for these comparisons. Therefore, the network meta-analyses may be considered as very preliminary. However, its presentation seems justified since guidelines for further research can be derived from the challenges it helped to identify. For risk of bias see Figure 3.

Weight (BMI) was chosen as an outcome criterion. This is justified, as the BMI is closely related to acute illness severity and long-term outcome in AN. Furthermore, it can be measured objectively (1, 14). However, several other aspects can be considered relevant in AN like overall eating disorder symptom severity, depressive symptoms or quality of life. It was not possible to compare treatments according to these aspects, due to insufficient data and a broad range of measures used. Furthermore, we could not differentiate between female and male patients with AN, as studies did not report on outcomes for both groups separately.

Furthermore, it is important to mention that there is a fundamental concern regarding the definition of “evidence based treatments” in psychotherapy research, which is based on a comparison of therapeutic approaches in RCTs.: Psychotherapeutic interventions (the “techniques”) explain only 15% of the variance in outcome (76). There is a range of further factors, especially patient and therapist variables that have a considerable impact (73).

### Recommendations for further research

The efficacy of new interventions should be compared to TAU conditions which have to be clearly described and to be as equivalent as possible in terms of dose of therapy, training and supervision (72). Furthermore, there is a need for the replication of findings. A most recent trial did exactly this: Comparing MANTRA, CBT-E, and SSCM (77). Additionally, the logic behind network meta-analyses should guide the planning of further trials: Any new trial should comprise at least one treatment arm with an effective “standard” intervention (family based treatment, CBT-E, FPT, SSCM, MANTRA) in order to link new/modified interventions to the present evidence base. Future network meta-analyses would very much profit from investigations directly comparing MANTRA with FT_AN in adult samples or comparing FT_AN with any complex treatment in adolescent samples, because these comparisons are needed to fill gaps in the networks of indirect comparisons. Future trials should ideally be multi-center trials with large sample sizes to allow for subgroup analyses. More adaptive treatment strategies for subgroups of patients with AN might improve remission rates in future (for an example see (78)). Furthermore, patient assessment has to be comprehensive including all variables that can be considered relevant moderators of treatment outcome such as co-morbidity, illness duration, BMI at the beginning of treatment, patients preferences for a specific treatment, impulsivity and previous treatment experiences (e.g., (79, 80)). Studies will also have to control for therapist factors and address possible mediators of change, as well as the issue of gender (81). Finally, there is a growing body of evidence to support a stage model of illness, with poorer prognosis in patients with longer duration of illness (3). Therefore, future treatment programs should distinguish between different stages of the illness and aims of treatment related to these stages: weight stabilization in a situation of severe underweight and medical instability, further weight gain until a normal weight range is reached, or relapse prevention. This also includes severe and enduring AN (see for example (82)). Finally, identifying and addressing maintaining factors remains a major challenge. One attempt to address one of these factors (dysfunctional habits) can be seen in a recent study by Steinglass et al. (83).

## Conclusions

In summary, no long-term superiority of one specialized treatment for AN over another specialized treatment could be demonstrated. Adult and adolescent patients should be distinguished, as groups differ in terms of treatment approaches considered suitable as well as treatment response. Weight gains are larger in adolescents and more intense treatment settings.

## Author contributions

All authors contributed to the conception and design of the study. All except TB were involved in independent ratings of the included studies. AZ and AH organized the data base, AZ wrote the first draft of the manuscript. AH conducted the statistical analysis. All authors contributed to manuscript revision, read and approved the submitted version.

### Conflict of interest statement

The authors declare that the research was conducted in the absence of any commercial or financial relationships that could be construed as a potential conflict of interest.

## Footnotes

1 ICTRP, International Clinical Trials Registry Platform of the World Health Organization (WHO).
