# Supplementary material for: Psychotherapeutic Treatment for Anorexia Nervosa: A Systematic Review and Network Meta-Analysis
Source: Front Psychiatry. 2018 May 1;9:158. doi: 10.3389/fpsyt.2018.00158 (PMC5939188; doi:10.3389/fpsyt.2018.00158)
Supplement: Supplementary file 1 [file Table_1.docx]

**Supplement 1: Rating of study quality**

| **Quality rating** | **Criteria for RCT´s** | **Criteria for naturalistic studies** |
| --- | --- | --- |
| “high quality” | All quality criteria were met  Exception: in case of a drop-out rate > 20%, a possible bias due to drop-out was addressed and a reliable method to handle missing data was used | All criteria were met, except the criteria 7, 9 and 10 |
| “moderate quality” | Criteria 2, 4, 5, 6, 8, 9, 11, 12 and 13 met  A maximum of 1-3 further criteria were not met  Exception: in case of a missing consort statement in older studies (not meeting criterion 9) the patient flow including drop-outs had to be reported in detail | Criteria 2, 5, 6, 11, 12 and 13 were met  Drop-outs were reported. |
| “low quality” | Not fulfilling criteria of “moderate quality” and not falling into the category of “very low quality” | Not fulfilling criteria of “moderate quality” and not falling into the category of “very low quality” |
| “very low quality” | The criteria 4, 6, 8 and 9 were not met | The criteria 2, 5 and 6 were not met, no report on drop-outs |

Note: For criteria see methods section (search strategy and selection of studies).
