# Supplementary material for: Psychotherapeutic Treatment for Anorexia Nervosa: A Systematic Review and Network Meta-Analysis
Source: Front Psychiatry. 2018 May 1;9:158. doi: 10.3389/fpsyt.2018.00158 (PMC5939188; doi:10.3389/fpsyt.2018.00158)
Supplement: Supplementary file 2 [file Table_2.docx]

**Supplement 2: Excluded studies**

| **Main reason for exclusion** | **Excluded studies** |
| --- | --- |
|  |  |
| **RCTs** | |
| **Study on relapse prevention**  (N = 2 from recent search, N = 1 from previous meta-analysis) | Fichter et al. 2012, (1); Godart et al. 2012, (2) |
| **Mixed sample** (N = 8) | Dingemanns et al. 2014, (4); Salbach-Andrae et al. 2009, (5); Simon et al. 2013, (6); Stein et al. 2013, (7); Steinglass et 2014, (8); Vocks et al. 2011, (9); Parling et al. 2016, (10); Kong et al. 2005, (11) |
| **No data on weight change** (N = 1) | Brockmeyer et al. 2014, (12) |
| **Very low quality**  (N = 3 from recent search, N = 13 from previous meta-analysis) | Hibbs et al. 2015, (13); Rhodes et al. 2008, (14); Whitney et al. 2012, (15) |
| **Other reasons**: Adaptive treatment approach, not comparable with other studies; sample size not sufficient to answer research question (N = 1) | Lock et al. 2015, (29) |
| **Studies from previous meta-analysis:** N = 14  (very low quality, study on relapse prevention)  Pike et al. 2003 (3), Bachar et al. 1999 (16); Ball & Mitchell 2004 (17), Bergh et al. 2002 (18), Channon et al. 1989 (19), Eckert et al. 1979 (20), Geist et al. 2000(21), Goldfarb et al. 1987 (22), Le Grange et al. 1992 (23), Pillay & Crisp 1981 (24), Russell et al. 1987 (25), Serfaty et al. 1999 (26), Wallin et al. 2000 (27), Weizmann 1985 (28) | |
| **Naturalistic studies** | |
| **Mixed sample** (N = 14) | Carter et al. 2009, (30); Marco et al. 2013, (31); Juarascio et al. 2013, (32); Girz et al. 2013, (33); Crino & Djokvucic 2010, (34); Hoste et al. 2015, (35); Henderson et al. 2014, (36); Schaffner & Buchanan 2008, (37); Begin et al. 2013, (38); Exerkate et al. 2009, (39); Johnston et al. 2015, (40); Schnicker et al. 2013, (41); Turner et al. 2015, (42); Dancyger et al. 2003, (43) |
| **No data on weight change; mixed sample**  (N = 6) | Ben-Porath et al. 2010, (44); Ornstein et al. 2012, (45); Jones et al. 2007, (46); Olmsted et al. 2013, (47); Byrne et al. 2011, (48); Paulson-Karlson et al. 2009, (49) |
| **Second time point of measurement not within a time frame of three years** (N = 1) | Amemiya et al. 2012, (50) |
| **No arm on psychotherapy** (N = 1) | Del Valle et al. 2010, (51) |
| **Sample size < 30** (N = 2) | Chen et al. 2015, (52), Goldstein et al. 2011, (53) |
| **Very low quality** (N = 3) | Ashley & Crino 2010, (54), O´Reilly et al. 2014, (55), Gentile et al. 2008, (56) |
| **Other reasons** (N = 3)  (No differentiation between treatment settings, archival data; only survival curves reported; data already included from a previous publication of the study) | Freudenberg et al. 2016 (57), Accurso et al. 2015, (100), Calugi et al. 2015, (58) |
| **Excluded naturalistic studies from the previous meta-analysis**: (N = 41)  (sample size < N = 30, very low quality, mixed sample, no treatment arm on psychotherapy or no data on weight change):  Bossert et al. 1987 (59), Brambilla et al. 1995 (60), Danziger et al, 1989 (61), Deep-Soboslay et al. 2000 (62), Gerlinghoff et al. 1991 (63), Herzog et al. 1996 (64), Herzog et al. 2004 (65), Okamoto et al. 2002 (66), Pertschuk et al 1978 (67), Pertschuk et al. 1981 (68), Waller et al 2003 (69), Zeeck et al. 2006 (70), Geller et al. 2005 (71), Santonastaso et al. 2001 (72), Ro et al. 2004 (73), Vandereycken et al, 1978 (74), Piran et al, 1989 (75), Shugar et al. 1995 (76), Gerlinghoff et al. 1998 (77), Andersen et al. 1996 (78), Barbarich et al. 2004 (79), Fassino et al. 2001 (80), Szabo et al, 1998 (81), Moser et al. 2003 (82), Manara et al. 2005 (83), Jacobi et al. 1997 (84), Lauer et al. 1999 (85), Bean et al. 2004 (86), Bennighoven et al. 2006 (87), Calvo Sagardoy et al. 1989 (88), Griffith et al. 1997 (89), Nussbaum et al. 1985 (90), Steiner et al. 1990 (91), Sutandar Pinnock et al. 2003 (92), Thomä 1961 (93), Treat et al. 2005 (94), Stonehill et al. 1977 (95), Zeeck et al. 2005 (96), Bloks et al. 2001 (97), Carter et al. 2004 (98), Ostuzzi et al. 1999 (99) | |

1. Fichter MM, Quadflieg N, Nisslmuller K, Lindner S, Osen B, Huber T, Wunsch-Leiteritz W. Does internet-based prevention reduce the risk of relapse for anorexia nervosa? *Behav Res Ther* (2012) **50**:180–90. doi:10.1016/j.brat.2011.12.003

2. Godart N, Berthoz S, Curt F, Perdereau F, Rein Z, Wallier J, Horreard AS, Kaganski I, Lucet R, Atger F, et al. A randomized controlled trial of adjunctive family therapy and treatment as usual following inpatient treatment for anorexia nervosa adolescents. *PLoS One* (2012) **7**:e28249. doi:10.1371/journal.pone.0028249

3. Pike KM, Walsh BT, Vitousek K, Wilson GT, Bauer J. Cognitive behavior therapy in the posthospitalization treatment of anorexia nervosa. *Am J Psychiatry* (2003) **160**:2046–2049. doi:10.1176/appi.ajp.160.11.2046

4. Dingemans AE, Danner UN, Donker JM, Aardoom JJ, van Meer F, Tobias K, van Elburg AA, van Furth EF. The Effectiveness of Cognitive Remediation Therapy in Patients with a Severe or Enduring Eating Disorder: A Randomized Controlled Trial. *Psychother Psychosom* (2014) **83**:29–36. doi:10.1159/000355240

5. Salbach-Andrae H, Bohnekamp I, Bierbaum T, Schneider N, Thurn C, Stiglmayr C, Lenz K, Pfeiffer E, Lehmkuhl U. Dialectical behavior therapy and cognitive behavior therapy for anorexia and bulimia nervosa among adolescents: A randomized, controlled trial with a waiting control group. *Kindh Entwickl* (2009) **18**:180–90.

6. Simon W, Lambert MJ, Busath G, Vazquez A, Berkeljon A, Hyer K, Granley M, Berrett M. Effects of providing patient progress feedback and clinical support tools to psychotherapists in an inpatient eating disorders treatment program: a randomized controlled study. *Psychother Res* (2013) **23**:287–300. doi:10.1080/10503307.2013.787497

7. Stein KF, Corte C, Chen DG, Nuliyalu U, Wing J. A randomized clinical trial of an identity intervention programme for women with eating disorders. *Eur Eat Disord Rev* (2013) **21**:130–42. doi:10.1002/erv.2195

8. Steinglass JE, Albano AM, Simpson HB, Wang Y, Zou J, Attia E, Walsh BT. Confronting fear using exposure and response prevention for anorexia nervosa: A randomized controlled pilot study. *Int J Eat Disord* (2014) **47**:174–80. doi:10.1002/eat.22214

9. Vocks S, Schulte D, Busch M, Gronemeyer D, Herpertz S, Suchan B. Changes in neuronal correlates of body image processing by means of cognitive-behavioural body image therapy for eating disorders: a randomized controlled fMRI study. *Psychol Med* (2011) **41**:1651–63. doi:10.1017/s0033291710002382

10. Parling T, Cernvall M, Ramklint M, Holmgren S, Ghaderi A. A randomised trial of Acceptance and Commitment Therapy for Anorexia Nervosa after daycare treatment, including five-year follow-up. *BMC Psychiatry* (2016) **16**: doi:10.1186/s12888-016-0975-6

11. Kong S. Day treatment programme for patients with eating disorders: randomized controlled trial. *J Adv Nurs* (2005) **51**:5–14. doi:10.1111/j.1365-2648.2005.03454.x

12. Brockmeyer T, Ingenerf K, Walther S, Wild B, Hartmann M, Herzog W, Bents H, Friederich HC. Training cognitive flexibility in patients with anorexia nervosa: a pilot randomized controlled trial of cognitive remediation therapy. *Int J Eat Disord* (2014) **47**:24–31. doi:10.1002/eat.22206

13. Hibbs R, Magill N, Goddard E, Rhind C, Raenker S, Macdonald P, Todd G, Arcelus J, Morgan J, Beecham J, et al. Clinical effectiveness of a skills training intervention for caregivers in improving patient and caregiver health following in-patient treatment for severe anorexia nervosa: pragmatic randomised controlled trial. *Br J Psychiatry Open* (2015) **1**:56–66. doi:10.1192/bjpo.bp.115.000273

14. Rhodes P, Baillee A, Brown J, Madden S. Can parent-to-parent consultation improve the effectiveness of the Maudsley model of family-based treatment for anorexia nervosa? A randomized controlled trial. *J Fam Ther* (2008) **30**:96–108.

15. Whitney J, Murphy T, Landau S, Gavan K, Todd G, Whitaker W, Treasure J. A practical comparison of two types of family intervention: an exploratory RCT of family day workshops and individual family work as a supplement to inpatient care for adults with anorexia nervosa. *Eur Eat Disord Rev* (2012) **20**:142–50. doi:10.1002/erv.1076

16. Bachar E, Latzer Y, Kreitler S, Berry EM. Empirical comparison of two psychological therapies. Self psychology and cognitive orientation in the treatment of anorexia and bulimia. *J Psychother Pract Res* (1999) **8**:115–128.

17. Ball J, Mitchell P. A Randomized Controlled Study of Cognitive Behavior Therapy and Behavioral Family Therapy for Anorexia Nervosa Patients. *Eat Disord* (2004) **12**:303–314. doi:10.1080/10640260490521389

18. Bergh C, Brodin U, Lindberg G, Sodersten P. Randomized controlled trial of a treatment for anorexia and bulimia nervosa. *Proc Natl Acad Sci* (2002) **99**:9486–9491. doi:10.1073/pnas.142284799

19. Channon S, de Silva P, Hemsley D, Perkins R. A controlled trial of cognitive-behavioural and behavioural treatment of anorexia nervosa. *Behav Res Ther* (1989) **27**:529–535.

20. Eckert E, Goldberg S, Halmi K, Casper R, Davis J. Behaviour therapy in anorexia nervosa. *Br J Psychiatry* (1979) **134**:55–59.

21. Geist R, Heinmaa M, Stephens D, Davis R, Katzman D. Comparison of family therapy and family group psychoeducation in adolescents with anorexia nervosa. *Can J Psychiatry* (2000) **45 (2)**:173–178.

22. Goldfarb LA, Fuhr R, Tsujimoto RN, Fischman SE. Systematic desensitization and relaxation as adjuncts in the treatment of anorexia nervosa: a preliminary study. *Psychol Rep* (1987) **60**:511–518. doi:10.2466/pr0.1987.60.2.511

23. Le Grange D, Eisler I. Evaluation of family treatments in adolescent anorexia nervosa. *Int J Eat Disord* (1992) **12**:347–357.

24. Pillay M, Crisp AH. The impact of social skills training within an established in-patient treatment programme for anorexia nervosa. *Br J Psychiatry J Ment Sci* (1981) **139**:533–539.

25. Russell G, Szmukler G, Dare C, Eisler I. An evaluation of family therapy in anorexia nervosa and bulimia nervosa. *Arch Gen Psychiatry* (1987) **44**:1047–1056.

26. Serfaty M, Turkington D, Heap M, Ledsham L, Jolley E. Cognitive therapy versus dietary counselling in the outpatint treatment of anorexia nervosa: effects of the treatment phase. *Eur Eat Disord Rev* (1999)334–350.

27. Wallin U, Kronovall P, Majewski M-L. Body awareness therapy in teenage anorexia nervosa: outcome after 2 years. *Eur Eat Disord Rev* (2000) **8**:19–30.

28. Weizman A, Tyano S, Wijsenbeck H, Ben David M. Behaviour therapy, pimozid treatment and prolactin secretion in anorexia nervosa. *Psychother Psychosom* (1985) **43**:136–140.

29. Lock J, Le Grange D, Agras WS, Fitzpatrick KK, Jo B, Accurso E, Forsberg S, Anderson K, Arnow K, Stainer M. Can adaptive treatment improve outcomes in family-based therapy for adolescents with anorexia nervosa? Feasibility and treatment effects of a multi-site treatment study. *Behav Res Ther* (2015) **73**:90–5. doi:10.1016/j.brat.2015.07.015

30. Carter JC, McFarlane TL, Bewell C, Olmsted MP, Woodside DB, Kaplan AS, Crosby RD. Maintenance treatment for anorexia nervosa: a comparison of cognitive behavior therapy and treatment as usual. *Int J Eat Disord* (2009) **42**:202–7. doi:10.1002/eat.20591

31. Marco JH, Perpina C, Botella C. Effectiveness of cognitive behavioral therapy supported by virtual reality in the treatment of body image in eating disorders: one year follow-up. *Psychiatry Res* (2013) **209**:619–25. doi:10.1016/j.psychres.2013.02.023

32. Juarascio A, Shaw J, Forman E, Timko CA, Herbert J, Butryn M, Bunnell D, Matteucci A, Lowe M. Acceptance and commitment therapy as a novel treatment for eating disorders: an initial test of efficacy and mediation. *Behav Modif* (2013) **37**:459–89. doi:10.1177/0145445513478633

33. Girz L, Robinson A, Foroughe M, Jasper K, Boachie A. Adapting family-based therapy to a day hospital programme for adolescents with eating disorders: preliminary outcomes and trajectories of change. *J Fam Ther* (2013) **35 (Suppl. 1)**:102–120.

34. Crino N, Djokvucic I. Cohesion to the group and its association with attendance and early treatment response in an adult day-hospital program for eating disorders: A preliminary clinical investigation. *Clin Psychol* (2010) **14**:54–61.

35. Hoste R. Incorporating family-based therapy principles into a partial hospitalization programme for adolescents with anorexia nervosa: Challenges and considerations. *J Fam Ther* (2015) **37**:41–60.

36. Henderson K, Buchholz A, Obeid N, Mossiere A, Maras D, Norris M, Harrison M, Feder S, Spettigue W. A Family-Based Eating Disorder Day Treatment Program for Youth: Examining the Clinical and Statistical Significance of Short-Term Treatment Outcomes. *Eat Disord* (2014) **22**:1–18. doi:10.1080/10640266.2014.857512

37. Schaffner AD, Buchanan LP. Integrating Evidence-Based Treatments with Individual Needs in an Outpatient Facility for Eating Disorders. *Eat Disord* (2008) **16**:378–392. doi:10.1080/10640260802370549

38. Bégin C, Gagnon-Girouard M-P, Aimé A, Ratté C. Trajectories of Eating and Clinical Symptoms Over the Course of a Day Hospital Program for Eating Disorders. *Eat Disord* (2013) **21**:249–264. doi:10.1080/10640266.2013.779188

39. Exterkate CC, Vriesendorp PF, de Jong CAJ. Body attitudes in patients with eating disorders at presentation and completion of intensive outpatient day treatment. *Eat Behav* (2009) **10**:16–21. doi:10.1016/j.eatbeh.2008.10.002

40. Johnston JAY, O’Gara JSX, Koman SL, Baker CW, Anderson DA. A Pilot Study of Maudsley Family Therapy With Group Dialectical Behavior Therapy Skills Training in an Intensive Outpatient Program for Adolescent Eating Disorders: Family Therapy and Dialectical Behavior Therapy. *J Clin Psychol* (2015) **71**:527–543. doi:10.1002/jclp.22176

41. Schnicker K, Legenbauer T, Hiller W. Therapy Effects and Response Rates of Patients with Eating Disorders: A Naturalistic Study. *Verhaltenstherapie* (2011) **21**:31–38. doi:10.1159/000324220

42. Turner H, Marshall E, Stopa L, Waller G. Cognitive-behavioural therapy for outpatients with eating disorders: Effectiveness for a transdiagnostic group in a routine clinical setting. *Behav Res Ther* (2015) **68**:70–75. doi:10.1016/j.brat.2015.03.001

43. Dancyger I, Fornari V, Schneider M, Fisher M, Frank S, Goodman B, Sison C, Wisotsky W. Adolescents and eating disorders: an examination of a day treatment program. *Eat Weight Disord EWD* (2003) **8**:242–248.

44. Ben-Porath D, Wisniewski L, Warren M. Outcomes of a day treatment program for eating disorders using clinical and statistical significance. *J Contemp Psychother* (2010) **40**:115–123.

45. Ornstein RM, Lane-Loney SE, Hollenbeak CS. Clinical outcomes of a novel, family-centered partial hospitalization program for young patients with eating disorders. *Eat Weight Disord EWD* (2012) **17**:e170-177.

46. Jones A, Bamford B, Ford H, Schreiber-Kounine C. How important are motivation and initial Body Mass Index for outcome in day therapy services for eating disorders? *Eur Eat Disord Rev* (2007) **15**:283–289. doi:10.1002/erv.736

47. Olmsted MP, McFarlane T, Trottier K, Rockert W. Efficacy and intensity of day hospital treatment for eating disorders. *Psychother Res* (2013) **23**:277–286. doi:10.1080/10503307.2012.721937

48. Byrne SM, Fursland A, Allen KL, Watson H. The effectiveness of enhanced cognitive behavioural therapy for eating disorders: an open trial. *Behav Res Ther* (2011) **49**:219–26. doi:10.1016/j.brat.2011.01.006

49. Paulson-Karlsson G, Engstrom I, Nevonen L. A pilot study of a family-based treatment for adolescent anorexia nervosa: 18- and 36-month follow-ups. *Eat Disord* (2009) **17**:72–88. doi:10.1080/10640260802570130

50. Amemiya N, Takii M, Hata T, Morita C, Takakura S, Oshikiri K, Urabe H, Tokunaga S, Nozaki T, Kawai K, et al. The outcome of Japanese anorexia nervosa patients treated with an inpatient therapy in an internal medicine unit. *Eat Weight Disord* (2012) **17**:e1-8. doi:10.3275/8034

51. del Valle MF, Perez M, Santana-Sosa E, Fiuza-Luces C, Bustamante-Ara N, Gallardo C, Villasenor A, Graell M, Morande G, Romo GR, et al. Does resistance training improve the functional capacity and well being of very young anorexic patients? A randomized controlled trial. *J Adolesc Health* (2010) **46**:352–8. doi:10.1016/j.jadohealth.2009.09.001

52. Chen EY, Segal K, Weissman J, Zeffiro TA, Gallop R, Linehan MM, Bohus M, Lynch TR. Adapting dialectical behavior therapy for outpatient adult anorexia nervosa--a pilot study. *Int J Eat Disord* (2015) **48**:123–32. doi:10.1002/eat.22360

53. Goldstein M, Peters L, Baillie A, McVeagh P, Minshall G, Fitzjames D. The effectiveness of a day program for the treatment of adolescent anorexia nervosa. *Int J Eat Disord* (2011) **44**:29–38. doi:10.1002/eat.20789

54. Ashley M, Crino N. A novel approach to treating eating disorders in a day-hospital treatment program. *Nutrition & Dietetics* (2010) **67**:155–159.

55. O’Reilly M, Carr C, Boylan C, Anglim M, Houlihan B. Anorexia nervosa (AN) in inpatients at a children’s hospital (2005-2011). *Ir Med J* (2014) **107**:53–5.

56. Gentile MG, Manna GM, Ciceri R, Rodeschini E. Efficacy of inpatient treatment in severely malnourished anorexia nervosa patients. *Eat Weight Disord EWD* (2008) **13**:191–197.

57. Freudenberg C, Jones RA, Livingston G, Goetsch V, Schaffner A, Buchanan L. Effectiveness of individualized, integrative outpatient treatment for females with anorexia nervosa and bulimia nervosa. *Eat Disord* (2016) **24**:240–254. doi:10.1080/10640266.2015.1090868

58. Calugi S, Dalle Grave R, Sartirana M, Fairburn CG. Time to restore body weight in adults and adolescents receiving cognitive behaviour therapy for anorexia nervosa. *J Eat Disord* (2015) **3**: doi:10.1186/s40337-015-0057-z

59. Bossert S, Schnabel E, Krieg JC, Molitor P, Kemper J, Berger M. [Concept of integrative inpatient-ambulatory therapy in patients with anorexia nervosa: a revised therapeutic approach]. *Psychother Psychosom Med Psychol* (1987) **37**:331–336.

60. Brambilla F, Draisci A, Peirone A, Brunetta M. Combined cognitive-behavioral, psychopharmacological and nutritional therapy in eating disorders. 1. Anorexia nervosa--restricted type. *Neuropsychobiology* (1995) **32**:59–63.

61. Danziger Y, Carel CA, Tyano S, Mimouni M. Is psychotherapy mandatory during the acute refeeding period in the treatment of anorexia nervosa? *J Adolesc Health Care Off Publ Soc Adolesc Med* (1989) **10**:328–331.

62. Deep-Soboslay A, Sebastiani LM, Kaye WH. Weight Gain With Anorexia Nervosa. *Am J Psychiatry* (2000) **157**:1526–1526. doi:10.1176/appi.ajp.157.9.1526

63. Gerlinghoff M, Backmund H, Angenendt J, Linington A. Ein Tagklinisches Therapiemodell fr psychosomatische Eást”rungen. [A day hospital model for treating psychosomatic eating disorders.]. *Verhaltenstherapie* (1991) **1**:61–65.

64. Herzog T, Hartmann A, Falk C. [Total symptom-oriented and psychodynamic concept in inpatient treatment of anorexia nervosa. A quasi-experimental comparative study of 40 admission episodes]. *Psychother Psychosom Med Psychol* (1996) **46**:11–22.

65. Herzog T, Zeeck A, Hartmann A, Nickel T. Lower targetsfor weekly weight gain lead to better results in inpatient treatment of anorexia nervosa. A pilot study. *Eur Eat Disord Rev* (2004) **12**:164–168.

66. Okamoto A, Yamashita T, Nagoshi Y, Masui Y, Wada Y, Kashima A, Arii I, Nakamura M, Fukui K. A behavior therapy program combined with liquid nutrition designed for anorexia nervosa. *Psychiatry Clin Neurosci* (2002) **56**:515–520. doi:10.1046/j.1440-1819.2002.01047.x

67. Pertschuk M, Edwards N, Pomerleau O. A multiple-baseline approach to behavioral intervention in anorexia nervosa. *Behav Ther* (1978) **9**:368–376.

68. Pertschuk MJ, Forster J, Buzby G, Mullen JL. The treatment of anorexia nervosa with total parenteral nutrition. *Biol Psychiatry* (1981) **16**:539–550.

69. Waller DA, Mugan MN, Morshed T, Stetnick J, Cummings M, Hynan LS. Three-Year Follow-up Study of Children and Adolescents with Anorexia Nervosa Initially Treated in a Continuum of Care Program. *Eat Disord* (2003) **11**:63–72. doi:10.1080/10640260390199262

70. Zeeck A, Hartmann A, Wetzler-Burmeister E, Wirsching M. Zum Vergleich station„rer und tagesklinischer Therapie bei Anorexia Nervosa. *Z Psychosom Med Psychother* (2006) **52 (2)**:190–203.

71. Geller J, Zaitsoff S, Srikameswaran S. Tracking readiness and motivation for change in individuals with eating disorders over the course of treatment. *Cogn Ther Res* (2005) **29**:611–625.

72. Santonastaso P, Friederici S, Favaro A. Sertraline in the treatment of restricting anorexia nervosa: an open controlled trial. *J Child Adolesc Psychopharmacol* (2001) **11**:143–150. doi:10.1089/104454601750284045

73. Rø O, Martinsen EW, Hoffart A, Rosenvinge JH. Short-term follow-up of adults with long standing anorexia nervosa or non-specified eating disorder after inpatient treatment. *Eat Weight Disord EWD* (2004) **9**:62–68.

74. Vandereycken W, Pieters G. Short-term weight restauration in anorexia nervosa through operant conditioning. *Scand J Behav Ther* (1978) **7**:221–236.

75. Piran N, Langdon L, Kaplan A, Garfinkel P. Evaluation of a day hospital for eating disorders. *Int J Eat Disord* (1989) **8**:523–532.

76. Shugar G, Krueger S. Aggressive family communication, weight gain, and improved eating attitudes during systemic family therapy for anorexia nervosa. *Int J Eat Disord* (1995) **17**:23–31.

77. Gerlinghoff M, Backmund H, Franzen U. Evaluation of a day treatment programme for eating disorders. *Eur Eat Disord Rev* (1998) **6**:96–106.

78. Andersen AE, Stoner SA, Rolls BJ. Improved eating behavior in eating-disordered inpatients after treatment: Documentation in a naturalistic setting. *Int J Eat Disord* (1996) **20**:397–403.

79. Barbarich NC, McConaha CW, Gaskill J, La Via M, Frank GK, Achenbach S, Plotnicov KH, Kaye WH. An open trial of olanzapine in anorexia nervosa. *J Clin Psychiatry* (2004) **65**:1480–1482.

80. Fassino S, Abbate Daga G, Amianto F, Leombruni P, Garzaro L, Rovera GG. Nonresponder anorectic patients after 6 months of multimodal treatment: predictors of outcome. *Eur Psychiatry J Assoc Eur Psychiatr* (2001) **16**:466–473.

81. Szabo CP. Tuberculosis and anorexia nervosa. *South Afr Med J Suid-Afr* (1998) **88**:275–276.

82. Moser DJ, Benjamin ML, Bayless JD, McDowell BD, Paulsen JS, Bowers WA, Arndt S, Andersen AE. Neuropsychological functioning pretreatment and posttreatment in an inpatient eating disorders program. *Int J Eat Disord* (2003) **33**:64–70. doi:10.1002/eat.10108

83. Manara F, Manara A, Todisco P. Correlation between psychometric and biological parameters in anorexic and bulimic patients during and after an intensive day hospital treatment. *Eat Weight Disord* (2005) **10**:236–44.

84. Jacobi C, Dahme B, Rustenbach S. Vergleich kontrollierter Psycho- und Pharmakotherapiestudien bei Bulimia und Anorexia nervosa. *Psychother Psychosom Med Psychol* (1997) **47**:346–364.

85. Lauer CJ, Gorzewski B, Gerlinghoff M, Backmund H, Zihl J. Neuropsychological assessments before and after treatment in patients with anorexia nervosa and bulimia nervosa. *J Psychiatr Res* (1999) **33**:129–138.

86. Bean P, Loomis CC, Timmel P, Hallinan P, Moore S, Mammel J, Weltzin T. Outcome variables for anorexic males and females one year after discharge from residential treatment. *J Addict Dis* (2004) **23**:83–94. doi:10.1300/J069v23n02_06

87. Bennighoven D, Jürgens E, Mohr A, Heberlein I, Kunzendorf S, Jantschek G. Different changes of body-images in patients with anorexia or bulimia nervosa during inpatient psychosomatic ttreatment. *Eur Eat Disord Rev* (2006) **14**:88–96.

88. Calvo Sagardoy R, Fernandez Ashton A, Ayuso Mateos JL, Bayon Perez C, Santo-Domingo Carrasco J. Between 5 and 9 years’ follow-up in the treatment of anorexia nervosa. *Psychother Psychosom* (1989) **52**:133–139.

89. Griffiths RA, Beumont PJV, Russell J, Touyz SW, Moore G. The Use of Guardianship Legislation for Anorexia Nervosa: A Report of 15 Cases. *Aust N Z J Psychiatry* (1997) **31**:525–531. doi:10.3109/00048679709065074

90. Nussbaum M, Shenker IR, Baird D, Saravay S. Follow-up investigation in patients with anorexia nervosa. *J Pediatr* (1985) **106**:835–840.

91. Steiner H, Mazer C, Litt IF. Compliance and outcome in anorexia nervosa. *West J Med* (1990) **153**:133–139.

92. Sutandar-Pinnock K, Blake Woodside D, Carter JC, Olmsted MP, Kaplan AS. Perfectionism in anorexia nervosa: A 6-24-month follow-up study. *Int J Eat Disord* (2003) **33**:225–229. doi:10.1002/eat.10127

93. Thomä H. *Anorexia nervosa. Geschichte, Klinik und Theorien der Pubertätsmagersucht (Engl.: Anorexia Nervosa, transl. G. Brydone. New York, International Universities Press, 1967)*. Bern, Stuttgart: Huber Klett (1961).

94. Treat TA, Gaskill JA, McCabe EB, Ghinassi FA, Luczak AD, Marcus MD. Short-term outcome of psychiatric inpatients with anorexia nervosa in the current care environment. *Int J Eat Disord* (2005) **38**:123–133. doi:10.1002/eat.20160

95. Stonehill E, Crisp AH. Psychoneurotic characteristics of patients with anorexia nervosa before and after treatment and at follow-up 4-7 years later. *J Psychosom Res* (1977) **21**:189–193.

96. Zeeck A, Hartmann A, Buchholz C, Herzog T. Drop out from inpatient treatment of anorexia nervosa. *Acta Psychiatr Scand* (2005) **111**:29–37.

97. Bloks H, Spinhoven P, Callewaert I, Willemse-Koning C, Turksma A. Changes in coping styles and recovery after inpatient treatment for severe eating disorders. *Eur Eat Disord Rev* (2001) **9**:397–415.

98. Carter JC, Blackmore E, Sutandar-Pinnock K, Woodside DB. Relapse in anorexia nervosa: a survival analysis. *Psychol Med* (2004) **34**:671–679. doi:10.1017/S0033291703001168

99. Ostuzzi R, Didonna F, Micciolo R. One-year weight follow-up in anorexia nervosa after inpatient psycho-nutritional rehabilitative treatment. *Eat Weight Disord EWD* (1999) **4**:194–197.

100. Accurso EC, Fitzsimmons-Craft EE, Ciao AC, Le Grange D. From efficacy to effectiveness: comparing outcomes for youth with anorexia nervosa treated in research trials versus clinical care. *Behav Res Ther* (2015) **65**:36–41. doi:10.1016/j.brat.2014.12.009
